# A late recurring and easily forgotten tumor: ovarian granulosa cell tumor

**DOI:** 10.1186/1477-7819-10-85

**Published:** 2012-05-16

**Authors:** Yi-Chan Chen, Liang-Che Chang, Ruey-Shyang Soong

**Affiliations:** 1 Division of general surgery, Department of surgery, 222, Mai-Chin Road, Keelung, Taiwan

## Abstract

Ovarian granulosa cell tumor (GCT) is a malignant tumor with slow progression. The recurrence of granulosa cell tumor often happens after 5 years, leading to a ‘forgotten tumor’ by the patient. We present the case of a 64-year-old woman with a presentation of left flank pain. An initial computed tomography scan revealed a single tumor with multiple adjacent organ invasions. Surgical intervention was prescribed and the pathological results revealed a metastatic granulosa cell tumor. We also review the literature for the follow-up and further management of this tumor.

## Introduction

Ovarian granulosa cell tumor (GCT) is a malignant tumor originating from the sex-cord stromal cells of the ovary. It is an uncommon primary malignant tumor of the ovary and represents 2% to 5% of all ovarian cancers [1]. This tumor is classified into juvenile GCT and adult GCT, and the majority of the cases are the adult type. GCT occurs at any age but there are two peaks in occurrence: at reproductive age and postmenopausal age [2]. The incidence of GCT in women in Western countries is twice that of women in Asia countries [3]. The clinical symptoms of GCT are abdominal pain and abnormal vaginal bleeding, and some cases may also present with menorrhagia, irregular menstruation, or amenorrhea in the reproductive age group. GCT is a cancer with long natural history, and recurrence often happens after 5 years of follow-up [1]. Most cases are diagnosed at stage I disease, therefore such patients need long-term follow-up.

## Case report

We present the case of a 64-year-old woman who visited our emergency ward due to left flank pain for 1 day. She had a history of hysterectomy and bilateral salpingo-oophorectomy 22 years ago, but could not remember the etiology of the primary disease due to the long time period and being lost to follow-up after the surgery. The pain was located on her left flank with radiation to the left lower quadrant of abdomen. The pain was dull in characteristic without aggravating factors. A physical examination revealed a 10 × 10 cm mass on the left upper quadrant of her abdomen without local tenderness.

**Figure 1:**
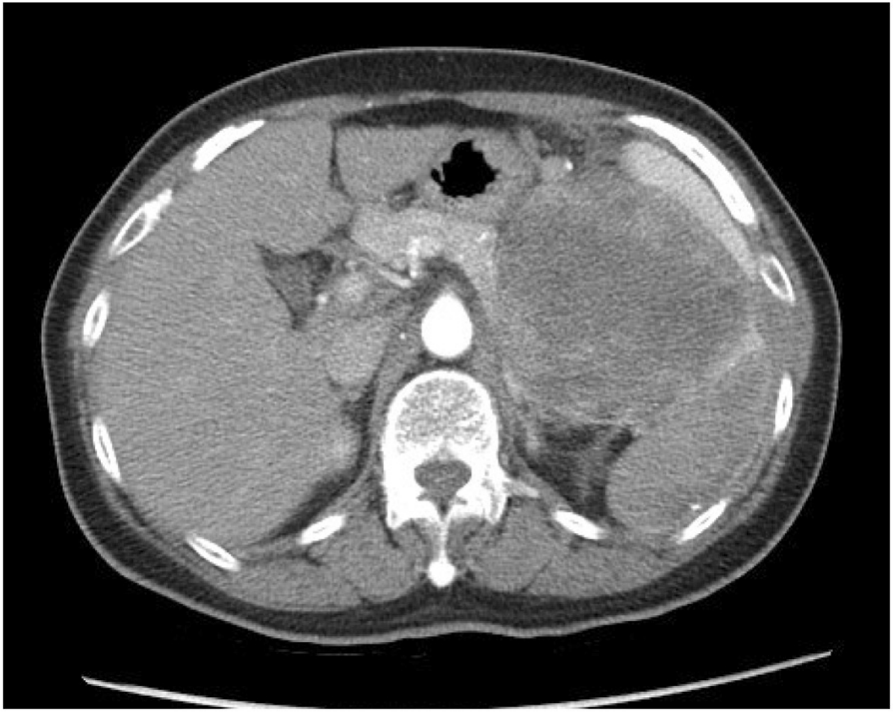
Abdominal computer tomography (CT) showing a single tumor measuring 20 × 15 cm in the perirenal space with spleen, pancreatic tail, and high gastric body invasion.

An 11.6 × 10.7 mass in the perirenal area was identified on ultrasonography. Abdominal computer tomography (CT) showed a single tumor measuring 10 × 10 cm in the perirenal space (Figure 1). The tumor was hypervascular on the arterial phase with possible gastric high body, spleen, and pancreatic tail invasion. The differential diagnosis was metastatic tumor with unknown origin and gastrointestinal stromal tumor of gastric origin. Maximal debulking surgery was performed including a splenectomy, distal pancreatecomy, partial left adrenectomy and partial excision of the diaphragm.

**Figure 2:**
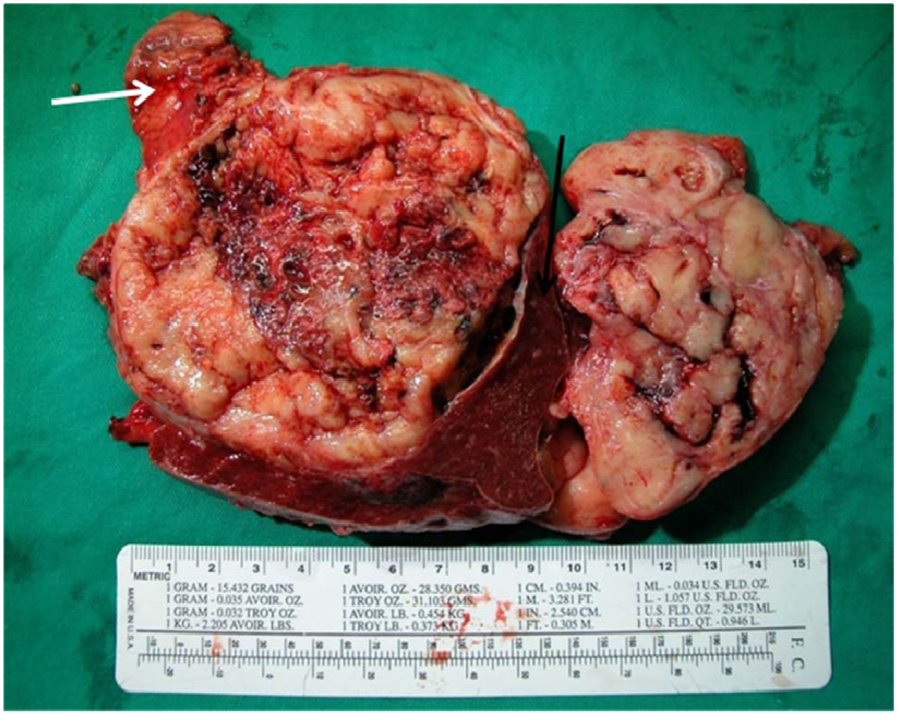
**The gross pathology of the tumor was well encapsulated, yellowish in color, and friable with multiple areas of hemorrhage and necrosis.** The black arrow shows the spleen; the white arrow is the pancreas tail.

**Figure 3:**
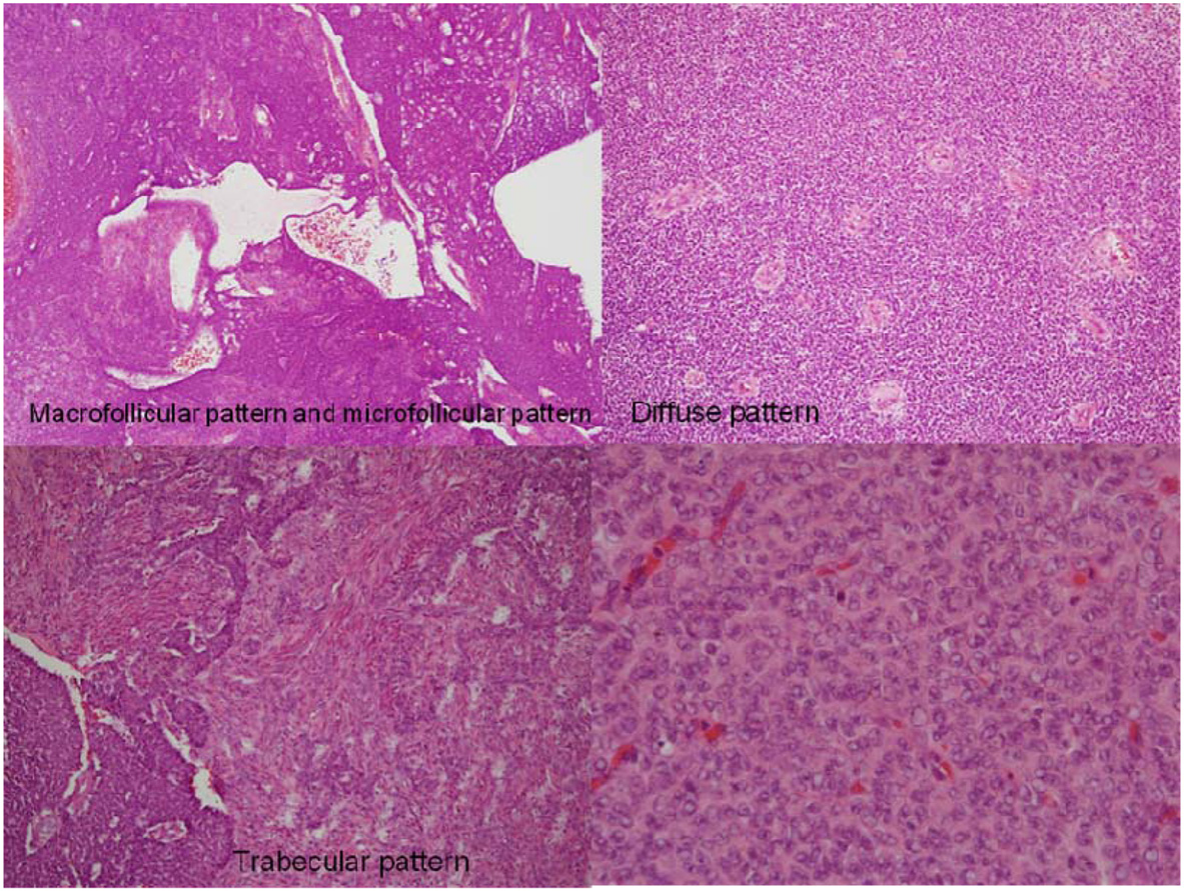
**Under hematoxylin and eosin stain, the tumor showed small round to oval cells with multiple distributive patterns, including macrofollicular, microfollicular, diffuse and trabecullar patterns.** The tumor cells also showed scanty cytoplasm with a coffee-bean-like nucleus.

**Figure 4:**
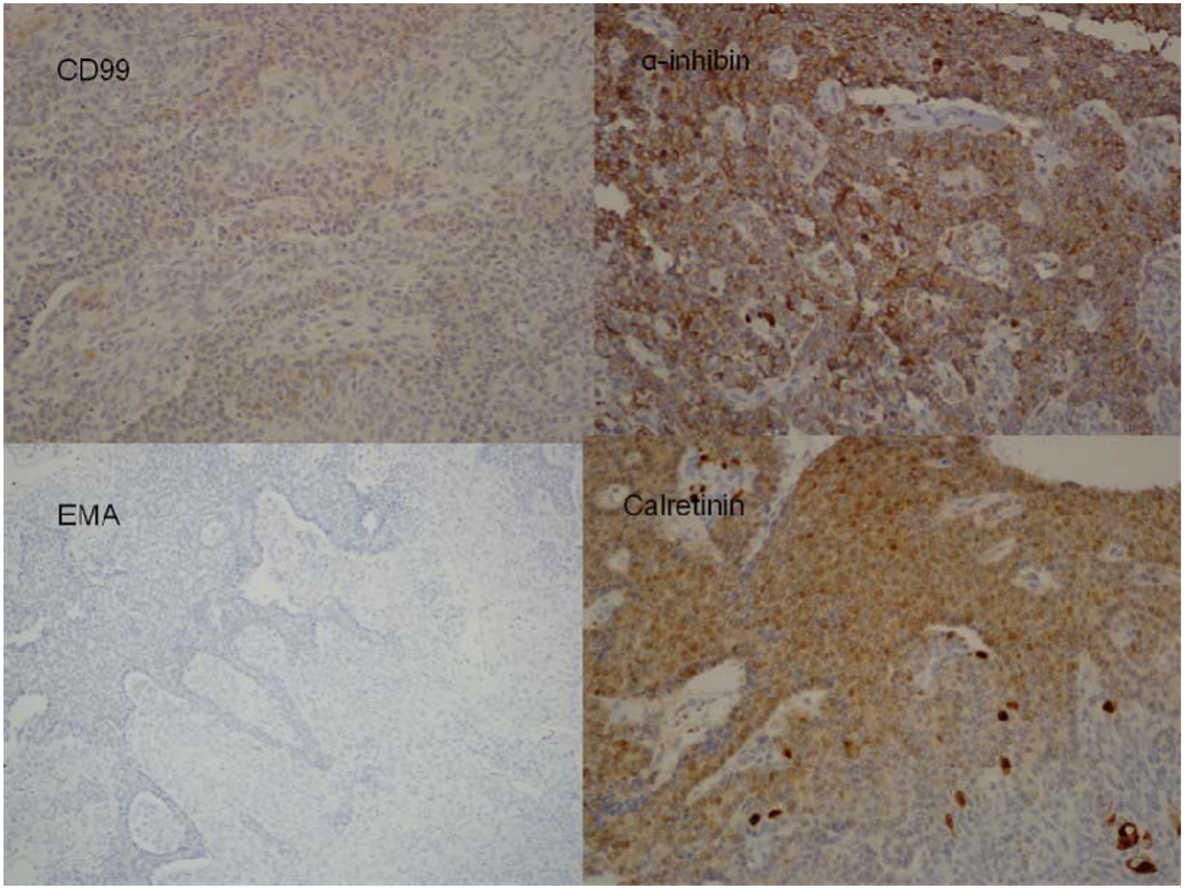
An immunohistological stain was positive for CD99, α-inhibin, calretinin (sex cord stromal tumor markers) and negative for epithelial membrane antigen (EMA) (sarcoma and mesenchymal tissue marker).

The gross findings of the tumor are shown in Figure 2. It was well encapsulated and yellowish in color. It was friable with multiple areas of hemorrhage and necrosis. The microscopic findings from a section of the tumor (Figure 3) showed small round to oval tumor cells with multiple distributive patterns, including macrofollicular, microfollicular, diffuse and trabecullar patterns. The tumor cells also showed scanty cytoplasm with a coffee-bean-like nucleus. An immunohistochemical (IHC) stain (Figure 4) was also performed to confirm diagnosis and the IHC stain was positive for CD99, α-inhibin and calretinin, and negative for epithelial membrane antigen (EMA). From the hematoxylin and eosin stain and the IHC stain results, the diagnosis of metastatic GCT was confirmed.

## Discussion

GCT is an uncommon malignant tumor of the sex cord/stromal tumor of the ovary. They only represent 2% to 5% of ovarian cancers but patients with GCT need long-term follow-up due to the slow growth of the tumor and long natural history of the tumor [1]. Recurrence of the tumor usually occurs almost 5 years after first treatment and the longest time of recurrence after treatment was 37 years [4]. The recurrence of our patient happened 22 years after the first treatment. Due to the long period of time point and forgotten history from the patient, the diagnosis of tumor recurrence was difficult to confirm before the surgery. The radiological findings of the GCT vary from solid mass to the cystic lesion and some may also present hemorrhage. The two most common classifications are multiseparated cystic mass and unlobulated solid mass with cystic portions [5].

The treatments for GCT include surgical management combined with further chemotherapy and radiation therapy. Because of the few cases, the surgical management is based on the treatment of other ovarian cancer. Although total abdominal hysterectomy and bilateral salpingo-oophorectomy is the standard treatment, for patients who desire pregnancy, unilateral salpingo-oophorectomy can be performed if the disease is only confined to one ovary. For patients with recurrent tumor, aggressive debulking surgery should be performed [6].

Adjuvant chemotherapy for GCT plays a beneficial role. Like other ovarian cancers, platinum-based and taxane-based chemotherapy should be considered first after surgical resection. Although patients with stage I GCT have an excellent prognosis and may not need chemotherapy, patients with a large tumor size, high-grade mytosis index and/or ruptured tumor are recommended for the chemotherapy [7]. For patients with recurrent GCT, chemotherapy should be prescribed to obtain better tumor control and long survival rate. Pectasides *et al*. reported combination chemotherapy with cisplatin, adriamycin and cyclophosphamide (CAP) for recurrent or advanced GCT. Five complete responses and one partial response were obtained [8].

Radiation therapy is also recommended for recurrent GCT, especially for patients with residual tumor after surgery. Based on the limited recurrent tumor or metastatic cases, some small series of intense radiation therapy for GCT have been reported to offer prolonged survival. Wolf *et al*. reported 14 patients with measurable GCT receiving radiation therapy after surgery. Complete response was observed in 6 of 14 patients (43%). Three of them relapsed 4 to 5 years later and the other three remain alive without recurrence for 10 to 21 years [9].

Hormonal therapy is also a treatment choice for recurrent GCT. Hardy *et al*. reported a review of the pathology for 22 GCT cases; all of the tumors were progesterone receptor related and 32% of them were estrogen receptor related. Several case reports also demonstrate treatment with hormonal therapy for recurrent GCT with complete or partial response [10].

## Conclusions

GCT has a low malignancy potential and long recurrence period. Patients with this disease at stage I usually do not need chemotherapy after surgery. Therefore, it would be an easily forgotten stromal cell tumor of the ovary if the patient is not regularly followed up. Patients with GCT require long-term follow-up since recurrence usually happens 5 years after first treatment [1]. The symptoms and signs are not specific for the diagnosis of recurrence. Estradiol and inhibin would be elevated before the recurrence of the GCT and can be used as markers during follow-up. Mullerian inhibitory substance (MIS) is still under investigation, but it could be a useful tumor marker of GCT activity [8]. However, because of the forgotten history by the patient and bizarre image findings, it usually leads to difficulty for clinicians to make an accurate diagnosis before surgical intervention. There is still no standard treatment for recurrent GCT, and maximal debulking is still the best strategy. Adjuvant radiation therapy and chemotherapy are still suggested, since benefits with regard to survival are reported. Radiation therapy should also be considered for the local control of possible microresidual tumors.

## Consent

Written informed consent was obtained from the patient for publication of this case report and any accompanying images. A copy of the written consent is available for review by the Editor-in-Chief of this journal.
